# Trends and socioeconomic inequalities in the dental attendance of adult smokers in Scotland from 2009 to 2019, a repeated cross-sectional study

**DOI:** 10.1186/s12889-024-19360-6

**Published:** 2024-08-08

**Authors:** Frances E. Warner, Andrea Sherriff, Shauna Culshaw, Richard Holliday, Vicky Ryan, David I. Conway

**Affiliations:** 1 School of Medicine, Dentistry and Nursing, University of Glasgow, Glasgow, UK; 2 School of Dental Sciences, Faculty of Medical Sciences, Newcastle University, Newcastle upon Tyne, UK; 3 Population Health Sciences Institute, Faculty of Medical Sciences, Newcastle University, Newcastle upon Tyne, UK

**Keywords:** Smoking, Dental attendance, Smoking cessation, Dental settings, Inequalities

## Abstract

**Background:**

Smoking continues to be the single largest cause of preventable disease and death and a major contributor to health inequalities. Dental professionals are well placed to offer behavioural support in combination with pharmacotherapy to increase smoking cessation rates across the population. We aimed to assess the trends and socioeconomic inequalities in the dental attendance of adult smokers in Scotland from 2009 to 2019 and examine the potential population reach of dental settings for smoking cessation interventions.

**Methods:**

A secondary analysis was conducted of combined Scottish Health Surveys (SHeS) from 2009/11, 2013/15 and 2017/19. ‘Recent’ dental attendance (within the past two years) was the focus and descriptive analysis examined attendance of self-reported smokers compared to non-smokers and stratified by the area-based Scottish Index of Multiple Deprivation (SIMD) and individual socioeconomic measures (income, education, and occupation). Generalised linear models were used to model recent attendance in non-smokers relative to smokers adjusted by the socioeconomic measures, for each of the survey cohorts separately. Absolute differences and risk ratios were calculated with 95% Confidence Intervals (CI).

**Results:**

Recent dental attendance was generally high and increased in both smokers (70–76%) and non-smokers (84–87%) from 2009/11 to 2017/19 and increased across all SIMD groups. After adjustment for sociodemographic variables, the adjusted Risk Difference (aRD) for recent attendance between non-smokers and smokers was 8.9% (95% CI 4.6%, 13.2%) by 2017/19. Within smokers, recent attendance was 7–9% lower in those living in the most deprived areas compared to those living in the least deprived areas over the three surveys.

**Conclusions:**

SHeS data from 2009 to 2019 demonstrated that a high and increasing proportion of smokers in the population attend the dentist, albeit slightly less frequently than non-smokers. There were large inequalities in the dental attendance of smokers, to a lesser extent in non-smokers, and these persisted over time. Dental settings provide a good potential opportunity to deliver population-level smoking cessation interventions, but smokers in the most deprived groups and older age groups may be harder to reach. Consideration should be given to ensure that these groups are given appropriate proportionate support to take up preventive interventions.

**Supplementary Information:**

The online version contains supplementary material available at 10.1186/s12889-024-19360-6.

## Background

Despite a notable reduction in smoking prevalence in recent decades, smoking continues to be the single largest cause of preventable disease and death worldwide [1] and thus remains a significant global public health threat [2]. Smoking remains a major contributor to health inequalities with social gradients showing those from the most deprived areas being five times more likely to smoke than those in the least deprived areas in Scotland [3, 4].

Smoking impacts on oral health as a known risk factor for periodontitis and oral cancers [5–8]. Additionally, it has been found to impact on the treatment of oral disease with the predictability and success of periodontal treatment notably reduced in smokers [9]. Current evidence suggests that smoking cessation is not only beneficial for periodontal treatment outcomes [10], but also reduces the risk of oral cancers and that this risk further declines as the time from cessation increases [11].

Smoking cessation services have been shown to aid the reduction in smoking rates [12–14] and given the severe consequences smoking has on oral health, it is appropriate for dental care professionals to play a role in this by providing evidence-based advice and referral to these services [15]. A recent Cochrane systematic review concluded that there was moderate-certainty evidence that smoking cessation rates increase if dental professionals offer behavioural support in combination with pharmacotherapy [16]. Additionally, attending the dentist can aid prevention and early detection of oral diseases that are more prevalent in smokers [15].

In the UK, an on-going large multi-centred randomised controlled trial is assessing the effectiveness of different approaches to smoking cessation including brief intervention, electronic cigarettes, and nicotine replacement therapy in dental settings upon quit rates [17]. Dental settings have previously been identified as potentially valuable locations to provide access to representative populations for public health and health improvement interventions [18–20]. Dental attendance has been shown to be lower among smokers compared to non-smokers, however, recent trends and socioeconomic inequalities in the Scottish adult population have not been examined in detail [21, 22]. We aimed to assess the trends and socioeconomic inequalities in the dental attendance of adult smokers in Scotland from 2009 to 2019 and examine the potential population reach of dental settings for smoking cessation interventions.

## Methods

### Data source

The Scottish Health Survey (SHeS) is a cross-sectional study that has been conducted annually by a consortium led by the Scottish Centre for Social Research (ScotCen) since 2008 and provides valuable information about the health of the Scottish population in private households. The nationally representative survey consists of a set of core questions, measurements and varying modules relating to specific conditions and risk factors [23]. Due to the COVID-19 pandemic, the data collected in the 2020 survey (via telephone rather than home visits) were published as experimental statistics and their inclusion in study trend analysis is not recommended [24]. Therefore, for the purposes of this study, we decided to focus on the pre-pandemic time period of 2009 to 2019. Data were accessed via UK Data Service [23].

### Ethical approval

Ethical approval for the study was submitted to and approved by The University of Glasgow College of Medical Veterinary & Life Sciences Research Ethics Committee in February 2023 (Project No: 200,220,188).

### Sample

The SHeS uses a clustered, stratified, multi-stage sample design to gather a representative sample of the general population living in private households in Scotland. An initial random sample of addresses is drawn from the Postcode Address File (PAF), this is a comprehensive list of residential addresses in Scotland. This initial sample comprises four sample types: main (core) sample with biological measures, main (core) sample without biological measures, child boost screening sample and Health Board boost sample. Our study analysed the main (core) sample without biological measures as this sample were asked the dental health service module questions. Survey interviewers visited eligible households and collected data using methods including a main computer assisted interview (CAI), paper self-completion questionnaires, height and weight measurements, and if applicable, collected biological samples [25].

The ‘survey’ package in R studio was used for the analysis. The variables in the dataset that define the components of the complex survey design (e.g. strata, Primary Sampling Units, weights) were specified and the analysis was weighted, this accounted for the complex survey design and any clustering present [26]. The precision of the estimates is indicated by the 95% Confidence Intervals. Weighting of the data prior to our analysis accounted for non-response and for the different selection probabilities of individuals and addresses. The non-response weights are intended to adjust for: non-contact, whole household refusals, non-response of individuals within responding households and non-response to particular aspects of the survey. The technical reports published for each survey year provide full details of survey methods used [25]. Analysis was restricted to respondents for whom all data for the variables of interest were available, there was no imputation of missing values.

### Measures

The following measures were taken from the SHeS 2009/11, 2013/15 and 2017/19.

Outcome variable:

- Dental attendance: ‘About how long ago was last visit to the dentist’, 5 categories (Less than a year ago; More than one year, up to two years ago; More than two years, up to five years ago; Never been to the dentist). This question is asked in the survey every two years. Our analysis focused on recent attendance (attendance within the previous two years) as the main outcome measure. We also assessed the secondary outcome of five year attendance in our analysis.

Exposure variable:

- Smoking status: ‘Do you smoke cigarette nowadays?’, 2 categories (Yes, No).

Covariates:

- Sex: male and female.
- Age: 7 categories (16–24, 25–34, 35–44, 45–54, 55–64, 65–74, 75+).
- Deprivation group: Scottish Index of Multiple Deprivation quintiles (SIMD 1 is the most deprived 20% of areas and SIMD 5 is the least deprived). SIMD is an area based relative measure of deprivation across 6,976 small areas (data zones) in Scotland [27].
- Education: Highest educational level, 6 categories (No qualifications, Other school level, Standard grade/GCSE or equivalent, Higher grade/A level or equivalent, Higher National Certificate/Higher National Diploma or equivalent, Degree or higher).
- Income: Equivalised income quintiles, 5 categories. 5th Quintile is the bottom income group and 1st Quintile is the top income group.
- Occupation: National Statistics Socio-economic Classification (NS-SEC), 8 categories (Never worked or Long-term unemployed, Routine, Semi-routine, Lower supervisory, Small employers, Intermediate, Lower managerial, Higher managerial) [28].

We analysed combined year datasets for 2009/11, 2013/15 and 2017/19 rather than individual years meaning that larger sample sizes were available for the analysis, whilst still allowing examination of attendance trends over time. Specific combined survey weights for each dataset (cohort), also available from UK data service online, were used for the analysis [3].

### Statistical analysis

For each of the three cohorts, descriptive analysis explored recent and five year dental attendance overall, and by smoking status, age group, sex, SIMD, education, occupation, income and survey year. Formal analysis was conducted for each cohort with weighted Generalised Linear models.

Univariable analysis was conducted to assess the association between recent attendance and each explanatory variable. The association between recent attendance and smoking status was incrementally adjusted for in a series of Generalised Linear models beginning with an unadjusted model (Model 1) and cumulatively adding age and sex (Model 2), survey year and SIMD (Model 3), and the individual socioeconomic measures education, occupation and income (Model 4). Unadjusted and adjusted Risk Difference (RD) and Risk Ratio (RR) with 95% confidence intervals were obtained. Interaction tests were performed for both outcomes to assess if the relationship between attendance and smoking was modified by the different explanatory variables. The concordance statistic (C-index) for each model was recorded to assess the model performance. C-index values range from 0 to 1, a higher value indicates that the model can discriminate between low and high risk subjects and models with a C-index of 0.7 or above are considered adequate to discriminate between risk profiles [29]. The same modelling method was used for the five year attendance outcome. Statistical analysis was undertaken using R Studio V.4.2.3.

## Results

### Smoking trends

Smoking prevalence in the three cohorts decreased steadily from 25 to 17% over the study period (Table 1).

**Table 1:** Smoking status by combined survey year

Combined survey year	Smoking status % (*n*)	
	Non-smoker	Smoker
2009/11 (*n* = 4013)	75 (3012)	25 (1001)
2013/15 (*n* = 3656)	80 (2919)	20 (737)
2017/19 (*n* = 4165)	83 (3459)	17 (706)

n = Number; Weighted with combined-year survey weights

### Attendance of smokers

Descriptively, recent attendance increased between 2009/11 and 2013/15 for both non-smokers (84–88%) and smokers (70–78%) and remained at approximately the 2013/15 levels in 2017/19 (Table 2). The difference between smokers and non-smokers did not change appreciably over the decade under study. The five year attendance followed a similar pattern (Table 2).

**Table 2:** Recent and five year attendance by smoking status and combined survey year

Combined survey year	Smoking status	Recent attendance % (n)	Five year attendance % (*n*)
2009/11 (*n* = 4013)	Non-smoker (*n* = 3012) Smoker (*n* = 1001)	84 (2539) 70 (701)	90 (2717) 82 (824)
2013/15 (*n* = 3656)	Non-smoker (*n* = 2919) Smoker (*n* = 737)	88 (2562) 78 (577)	93 (2710) 90 (660)
2017/19 (*n* = 4165)	Non-smoker (*n* = 3459) Smoker (*n* = 706)	87 (3002) 76 (534)	93 (3234) 87 (612)

Recent attendance = Attendance within 2 years; n = Number; Weighted with combined-year survey weights

**Fig. 1:**
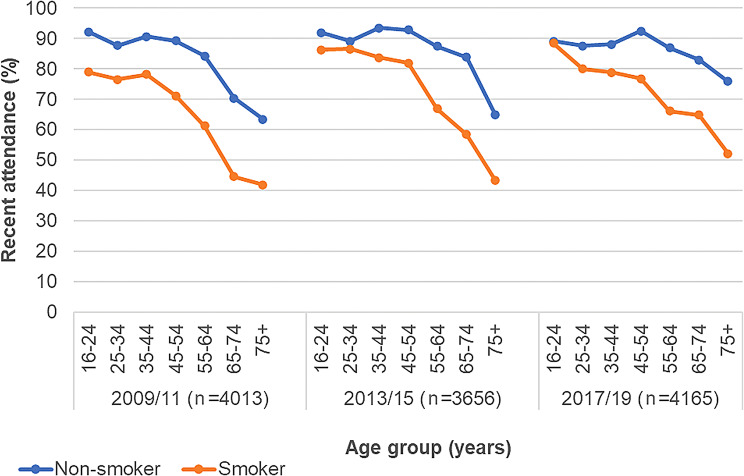
Recent attendance by smoking status and age group. Recent attendance = Attendance within 2 years

Recent attendance was on average higher for females, regardless of smoking status, it increased slightly in both sexes over time, notably in female smokers from 2009/11 to 2017/19 (74 to 82%). Recent attendance increased in each age group over time (Fig. 1), but in the over 55 groups there was a notable 18–26% difference between smokers and non-smokers and this was consistent over time (See Additional file 1: Supplementary Table 1).

### Socioeconomic inequalities in dental attendance

**Fig. 2:**
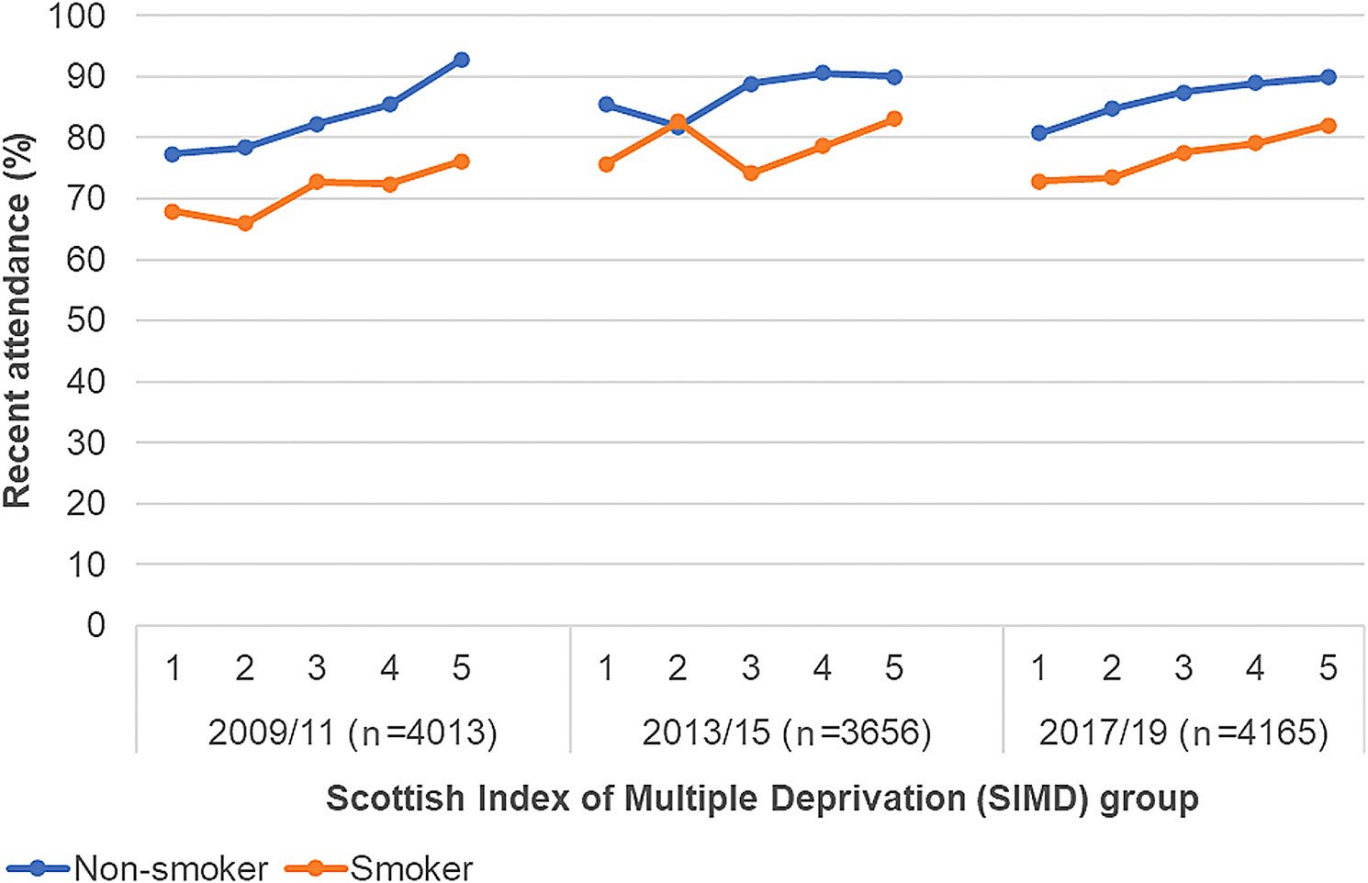
Recent attendance by smoking status and SIMD. Recent attendance = Attendance within 2 years; 1 = most deprived (SIMD 1); 5 = least deprived (SIMD 5)

Attendance increased in all SIMD groups but an inequality persisted with lower recent attendance rates in more deprived groups, regardless of smoking status, and this pattern repeated over the three time cohorts (Fig. 2). By 2017/19, 73% of smokers in SIMD 1 compared to 82% of smokers in SIMD 5 recently attended (See Additional file 1: Table 1). A similar pattern was evident for the individual socioeconomic measures with a persisting social gradient (See Additional file 1: Figures 1,2 and 3). For five year attendance of smokers the absolute inequality between SIMD 1 and 5 appeared to increase to 6% by 2017/19 (See Additional file 1: Table 1).

### Univariable and multivariable models for recent dental attendance and smoking

The unadjusted Risk Difference (RD) for recent attendance in non-smokers relative to smokers decreased over time from 14.3% (95% CI: 10.7, 17.9) in 2009/11 to 11.2% (95% CI: 7.0, 15.4) by 2017/19 (Table 3). After full adjustment for sociodemographic variables, the aRD decreased in all three cohorts by 1–2%, to 11.9% (95% CI: 8.3, 15.6) in 2009/11 and to 8.9% (95% CI: 4.6, 13.2) by 2017/19, signifying an improvement in attendance of smokers.

**Table 3:** Unadjusted and adjusted risk differences (95% CI) for recent dental attendance comparing non-smokers to smokers

Scottish Health Survey Year Recent attendance % (*n*)	Model 1 Univariable	Model 2 adjusted for age and sex	Model 3 adjusted as for Model 2 plus SIMD and survey year^a^	Model 4 adjusted as for Model 3 plus individual SES measures^b^
	RD % (95% CI)	aRD % (95% CI)	aRD % (95% CI)	aRD % (95% CI)
2009/11 (*n*= 4013)				
Smoker (ref) 70 (701)				
Non-smoker 84 (2539)	14.3 (10.7, 17.9) C-index 0.58 p value < 0.0001	16.5 (13.0, 20.0) C-index 0.71 p value < 0.0001	13.9 (10.3, 17.4) C-index 0.73 p value < 0.0001	11.9 (8.3, 15.6) C-index 0.74 p value < 0.0001
2013/15 (*n* = 3656)				
Smoker (ref) 78 (578)				
Non-smoker 88 (2562)	9.4 (5.6, 13.3) C-index 0.56 p value < 0.0001	11.1 (7.4, 14.8) C-index 0.70 p value < 0.0001	10.1 (6.5, 13.8) C-index 0.71 p value < 0.0001	8.4 (4.6, 12.2) C-index 0.73 p value < 0.0001
2017/19 (*n* = 4165)				
Smoker (ref) 76 (534)				
Non-smoker 87 (3002)	11.2 (7.0, 15.4)	12.3 (8.1, 16.4)	10.4 (6.1, 14.6)	8.9 (4.6, 13.2)
	C-index 0.56 p value < 0.0001	C-index 0.61 p value < 0.0001	C-index 0.67 p value < 0.0001	C-index 0.69 p value < 0.0001

Recent attendance = Attendance within 2 years; RD Risk Difference; aRD Adjusted Risk Difference; CI confidence interval; n number; C-index Concordance index; ^a^survey year (individual year within each cohort), quintiles of the Scottish Index of Multiple Deprivation; ^b^education, occupation, income; Weighted with combined-year survey weights

The C-index improved as each model was further adjusted with readings ranging from 0.69 to 0.77 for the fully adjusted models. Table 3 and Additional file 1: Fig. 4 shows the RD (and 95% CIs) from univariable and fully adjusted multivariable models for recent attendance of non-smokers relative to smokers. Additional file 1: Supplementary Table 2 displays Risk Ratios (and 95% CIs) for these models.

### Multivariable models for five year attendance and smoking

The aRD between non-smokers and smokers decreased in all 3 cohorts after fully adjusting for sociodemographic variables and was 6% by 2017/19 (95% CI: 2.7, 8.4). The findings of the univariable and multivariable analysis for five year attendance are presented in Additional file 1: Supplementary Tables 3 and 4. Interaction tests are displayed in Additional file 1: Supplementary Table 1 and suggested that there was generally no evidence of statistically significant interactions for both outcomes.

## Discussion

This study examined the dental attendance patterns of adult smokers by sociodemographic group in Scotland through the analysis of Scottish Health Survey data from three time cohorts. We found that smokers overall had a high level of recent attendance, an even higher level of attendance within five years. There was no evidence that socioeconomic deprivation changed the association between smoking and attendance.

Our descriptive analysis showed that differences in attendance between smokers and non-smokers did not appear to change across socioeconomic groups. However, the absolute inequality in attendance between the most and least deprived groups persisted over time, regardless of smoking status. When we adjusted for socioeconomic variables in our linear models this had only a slight effect on the Risk Differences or Risk Ratios, suggesting that the association between socioeconomic factors, smoking and dental attendance may be more complex.

It is not clear why there was a stark divergence of attendance rates by smoking status in over 55-year-old groups, but one possibility is this cohort having fewer natural teeth and as a result attending less. For example, 40% of those aged over 65 years were edentulous (had no teeth) in 2008/9 in Scotland [30]. Other studies have highlighted apparent low service access among older groups and this suggests a need for further research to explore reasons for this and identify approaches to increase attendance [21]. This pattern of lower attendance in older age-groups is also reported in routine administrative data on NHS dental service contacts [31].

A limitation of our study is that our data did not include information relating to the reason for attendance nor the past attendance history of individuals over time. For example, a recent attender in our study may be a smoker who attended for a ‘one off’ emergency appointment and has previously not attended for many years. Such infrequent contact with a dental professional may limit the potential opportunity for a smoking cessation intervention to be offered. Analysis of a similar nationally representative household survey for England noted that smokers were less likely to attend for a routine dental examination and more likely to attend symptomatically, i.e. for an emergency appointment, and this was regardless of deprivation [32]. They concluded that while dental teams have been shown to have an important impact on smoking cessation, contact with smokers within a dental setting may be limited and a common risk factor approach should be used across a range of health practitioners to ‘make every contact count’ [32]. Another notable limitation of the survey data, which has been echoed by other studies [33], is the self-reported nature of the variables for dental attendance, smoking status, education, income, and occupation. This may mean an underestimation of smoking prevalence occurs [34] and introduction of bias if some smokers misclassify themselves as non-smokers. Previous studies have been able to validate smoking status using salivary cotinine levels [35, 36] and some have indicated under-reporting of smoking by approximately 3% [36]. However, while the Scottish Health Survey does include an additional, albeit smaller, sample for whom biological cotinine measures are taken, this group were not asked the dental attendance question.

Official figures from the time period of interest in this study show that 94.2% of the Scottish population were registered with an NHS dentist as of 30 September 2018 and 69.9% of those had contact with a dentist in the previous two years [37]. However, this does not include private patients or unregistered patients (for example, attending for dental emergencies). We found that 85% of survey respondents self-reported dental attendance within the previous two years in the similar period (2017/19). The higher figure reported in our study was not unexpected as we relied upon self-reported data which could have resulted in over-estimation of attendance. Our results mentioned above are not dissimilar from the official statistics [31] which provides some external validity to the analysis. Some of the subgroups in our analysis still had small numbers despite using combined year datasets, and we acknowledge that the small numbers may affect the precision of the estimates. Ideally, we would have combined all the survey years of interest together to increase the sample size further and allow for effect modification to be assessed. However, this was not possible as we did not have access to the sampling framework and without this, we could not create our own combined survey weight for the analysis. Our study focused on the time period prior to COVID-19. Dental access was negatively impacted by the pandemic with one study reporting that after the dramatic fall in attendance due to lockdown measures there was a slow recovery to 64.8% of pre-pandemic levels by May 2022 in Scotland [38]. However, they did not specifically examine attendance of smokers and further research is required to examine the post-covid attendance levels of this group.

The problem of non-attendance or repeated missed healthcare appointments has been highlighted as an area of concern in the literature and studies have examined the issue in Scotland [39–41]. Both patient and practice factors were important when attendance patterns were assessed, with those aged 16–30, those over the age of 90 and low socioeconomic groups significantly more likely to miss multiple appointments [42]. Our study identified similar groups who may be ‘missed’ by interventions in dental settings, such as those aged over 55, and further research is currently being conducted to assess potential interventions to address repeated non-attendance [40].

Our study had several strengths, including the fact that the Scottish Health Survey has been conducted as an annual, nationwide survey since 2008. This allowed for analysis of population trends in Scotland over time and judgement on whether changes seen are real or related to sample size fluctuation. The combined datasets provided a larger sample than the individual years for analysis, increased the power of the study and allowed assessment of trends in attendance between different population groups over time. Other important strengths include the use of data from a single source meaning there was no reliance on linkage of datasets from multiple sources. The representativeness of the general population afforded by the sampling method strengthened the validity and aided the generalisability of our findings. However, it is important to acknowledge that declining survey response levels may jeopardise this [43] and only those residing in private households are included, which means the exclusion of some groups e.g. students in halls of residence [30]. It is also important to note that dental examinations in Scotland have been free for all National Health Service (NHS) patients since 2006, with any additional treatment required being costed for in a ‘fee per item’ system [44]. Additionally, some patients are exempt from all dental charges, such as those in receipt of certain benefit payments. Therefore, our results may not be generalisable to other countries that have different healthcare structures and where cost may be a larger barrier.

A further advantage of the dataset was the broad range of socioeconomic variables available, at both the area level and individual level, this allowed us to comprehensively detail how attendance varied across socioeconomic groups. Additionally, The Scottish Health Survey provides a wide range of high-quality data which allows measurement of health and behaviours at a population level [25, 36, 45].

## Conclusion

Between 2009 and 2019 there was a high and increasing proportion of smokers in the population attending the dentist, albeit slightly less frequently than non-smokers. There were large inequalities in the dental attendance of smokers, to a lesser extent in non-smokers, and these persisted over time. Dental settings provide a good potential opportunity to deliver population-level smoking cessation interventions, but there appears to be a small percentage of smokers in the most deprived groups and in older age groups who may be missed. Consideration should be given to ensure that those from more deprived socioeconomic groups are given appropriate proportionate support to the take up of preventive interventions including smoking cessation which are increasingly specified in evidence based clinical dental guidance [15]. In Scotland, the NHS dental contract changes, including an enhanced preventive advice and treatment payment item (specifying smoking cessation) along with an enhanced payment for patients resident in the most deprived (SIMD 1) could provide the additional support to deliver smoking cessation interventions reaching across the population [44].

### Electronic supplementary material

Below is the link to the electronic supplementary material.

Supplementary Material 1

## Data Availability

The datasets analysed during this study are available from the UK data archive:2009/11: https://beta.ukdataservice.ac.uk/datacatalogue/studies/study?id=7247: 10.5255/UKDA-SN-7247-52013/15: https://beta.ukdataservice.ac.uk/datacatalogue/studies/study?id=8100: 10.5255/UKDA-SN-8100-12017/19: https://beta.ukdataservice.ac.uk/datacatalogue/studies/study?id=8737: 10.5255/UKDA-SN-8737-1.

## Abbreviations

Risk: Difference
aRD: Adjusted Risk Difference
RR: Risk Ratio
aRR: Adjusted Risk Ratio
CI: Confidence Interval
c-index: Concordance statistic
N: Number
NHS: National Health Service
SES: Socioeconomic status
SHeS: Scottish Health Survey
SIMD: Scottish Index of Multiple deprivation
