# Supplementary material for: Trends and socioeconomic inequalities in the dental attendance of adult smokers in Scotland from 2009 to 2019, a repeated cross-sectional study
Source: BMC Public Health. 2024 Aug 8;24:2156. doi: 10.1186/s12889-024-19360-6 (PMC11308390; doi:10.1186/s12889-024-19360-6)
Supplement: Supplementary file 1 — Supplementary Material 1 [file 12889_2024_19360_MOESM1_ESM.docx]

**Supplementary Table 1. Recent and five year dental attendance of smokers and non-smokers by sociodemographic group**

| **Survey year** | **Sociodemographic variable** | **Smoking status** | **Recent attendance % (N)** | **5 year attendance % (N)** |
| --- | --- | --- | --- | --- |
| **2009/11**  **(N=4013)** | **-** | Non-smoker (N=3012)  Smoker (N=1001) | 84 (2539)  70 (701) | 90 (2717)  82 (824) |
| **2013/15 (N=3656)** | **-** | Non-smoker (N=2919)  Smoker (N=737) | 88 (2562)  78 (577) | 93 (2710)  90 (660) |
| **2017/19 (N=4165)** | **-** | Non-smoker (N=3459)  Smoker (N=706) | 87 (3002)  76 (534) | 93 (3234)  87 (612) |
|  | **Sex** |  |  |  |
| **2009/11** | Male (N=1956) | Non-smoker (N=1456)  Smoker (N=500) | 81 (1180)  66 (329) | 88 (1283)  78 (390) |
|  | Female (N=2057) | Non-smoker (N=1556)  Smoker (N=501) | 87 (1359)  74 (372) | 92 (1434)  87 (434) |
|  | Smoking*Sex |  | p value 0.55 | p value 0.12 |
| **2013/15** | Male (N=1769) | Non-smoker (N=1383)  Smoker (N=386) | 86 (1184)  71 (274) | 92 (1266)  86 (333) |
|  | Female (N=1886) | Non-smoker (N=1536)  Smoker (N=350) | 90 (1378)  87 (303) | 94 (1444)  93 (327) |
|  | Smoking*Sex |  | p value 0.0001 | p value 0.07 |
| **2017/19** | Male (N=2011) | Non-smoker (N=1643)  Smoker (N=368) | 84 (1376)  70 (257) | 93 (1520)  85 (311) |
|  | Female (N=2155) | Non-smoker (N=1817)  Smoker (N=338) | 89 (1626)  82 (277) | 94 (1714)  89 (302) |
|  | Smoking*Sex |  | p value 0.09 | p value 0.28 |
|  | **Age group** |  |  |  |
| **2009/11** | 16-24 (N=408) | Non-smoker (N=308)  Smoker (N=100) | 92 (284)  79 (79) | 97 (298)  94 (94) |
|  | 25-34 (N=677) | Non-smoker (N=480)  Smoker (N=197) | 88 (421)  77 (151) | 94 (452)  85 (168) |
|  | 35-44 (N=765) | Non-smoker (N=540)  Smoker (N=225) | 91 (490)  78 (176) | 95 (514)  88 (197) |
|  | 45-54 (N=777) | Non-smoker (N=579)  Smoker (N=198) | 89 (517)  72 (141) | 94 (545)  88 (174) |
|  | 55-64 (N=653) | Non-smoker (N=485)  Smoker (N=168) | 84 (409)  61 (103) | 89 (433)  75 (126) |
|  | 65-74 (N=428) | Non-smoker (N=345)  Smoker (N=83) | 70 (243)  45 (37) | 79 (272)  58 (48) |
|  | 75+ (N=307) | Non-smoker (N=276)  Smoker (N=31) | 63 (175)  42 (13) | 74 (204)  58 (18) |
|  | Smoking*Age |  | p value 0.20 | p value 0.10 |
| **2013/15** | 16-24 (N=382) | Non-smoker (N=287)  Smoker (N=95) | 92 (264)  86 (82) | 97 (278)  97 (93) |
|  | 25-34 (N=607) | Non-smoker (N=458)  Smoker (N=149) | 89 (408)  87 (129) | 97 (443)  95 (142) |
|  | 35-44 (N=615) | Non-smoker (N=480)  Smoker (N=135) | 94 (449)  84 (113) | 96 (461)  93 (126) |
|  | 45-54 (N=734) | Non-smoker (N=573)  Smoker (N=161) | 93 (532)  82 (132) | 96 (550)  91 (146) |
|  | 55-64 (N=596) | Non-smoker (N=481)  Smoker (N=115) | 88 (421)  67 (77) | 92 (443)  82 (94) |
|  | 65-74 (N=436) | Non-smoker (N=383)  Smoker (N=53) | 84 (321)  58 (31) | 89 (342)  77 (41) |
|  | 75+ (N=286) | Non-smoker (N=256)  Smoker (N=30) | 65 (166)  43 (13) | 75 (192)  60 (18) |
|  | Smoking*Age |  | p value 0.03 | p value 0.09 |
| **2017/19** | 16-24 (N=381) | Non-smoker (N=320)  Smoker (N=61) | 89 (285)  89 (54) | 97 (309)  100 (61) |
|  | 25-34 (N=713) | Non-smoker (N=558)  Smoker (N=155) | 88 (489)  80 (124) | 97 (541)  95 (147) |
|  | 35-44 (N=680) | Non-smoker (N=538)  Smoker (N=142) | 88 (474)  79 (112) | 96 (518)  90 (128) |
|  | 45-54 (N=783) | Non-smoker (N=632)  Smoker (N=151) | 92 (584)  77 (116) | 97 (611)  88 (133) |
|  | 55-64 (N=692) | Non-smoker (N=571)  Smoker (N=121) | 87 (497)  66 (80) | 92 (528)  77 (93) |
|  | 65-74 (N=550) | Non-smoker (N=496)  Smoker (N=54) | 83 (411)  65 (35) | 89 (439)  69 (37) |
|  | 75+ (N=368) | Non-smoker (N=345)  Smoker (N=23) | 76 (262)  52 (12) | 83 (288)  52 (12) |
|  | Smoking*Age |  | p value 0.17 | p value 0.001 |
|  | **SIMD group** |  |  |  |
| **2009/11** | SIMD 1 (N=735) | Non-smoker (N=436)  Smoker (N=299) | 77 (337)  68 (203) | 85 (372)  81 (242) |
|  | SIMD 2 (N=766) | Non-smoker (N=520)  Smoker (N=246) | 78 (408)  66 (162) | 86 (445)  80 (196) |
|  | SIMD 3 (N=834) | Non-smoker (N=632)  Smoker (N=202) | 82 (520)  73 (147) | 90 (570)  86 (173) |
|  | SIMD 4 (N=813) | Non-smoker (N=664)  Smoker (N=149) | 86 (568)  72 (108) | 91 (607)  85 (126) |
|  | SIMD 5 (N=866) | Non-smoker (N=761)  Smoker (N=105) | 93 (707)  76 (80) | 95 (724)  83 (87) |
|  | Smoking*SIMD |  | p value 0.78 | p value 0.58 |
| **2013/15** | SIMD 1 (N=632) | Non-smoker (N=426)  Smoker (N=206) | 85 (364)  76 (156) | 90 (382)  87 (180) |
|  | SIMD 2 (N=705) | Non-smoker (N=550)  Smoker (N=155) | 82 (450)  83 (128) | 87 (481)  91 (141) |
|  | SIMD 3 (N=673) | Non-smoker (N=514)  Smoker (N=159) | 89 (457)  74 (118) | 93 (479)  87 (139) |
|  | SIMD 4 (N=856) | Non-smoker (N=720)  Smoker (N=136) | 91 (652)  79 (107) | 96 (690)  92 (125) |
|  | SIMD 5 (N=791) | Non-smoker (N=708)  Smoker (N=83) | 90 (638)  83 (69) | 96 (677)  92 (76) |
|  | Smoking*SIMD |  | p value 0.07 | p value 0.15 |
| **2017/19** | SIMD 1 (N=765) | Non-smoker (N=536)  Smoker (N=229) | 81 (433)  73 (167) | 90 (483)  84 (192) |
|  | SIMD 2 (N=857) | Non-smoker (N=672)  Smoker (N=185) | 85 (569)  74 (136) | 91 (612)  83 (153) |
|  | SIMD 3 (N 827) | Non-smoker (N=693)  Smoker (N=134) | 87 (606)  78 (104) | 94 (652)  91 (122) |
|  | SIMD 4 (N=820) | Non-smoker (N=729)  Smoker (N=91) | 89 (649)  79 (72) | 94 (682)  93 (85) |
|  | SIMD 5 (N=896) | Non-smoker (N=829)  Smoker (N=67) | 90 (745)  82 (55) | 97 (805)  90 (60) |
|  | Smoking*SIMD |  | p value 0.98 | p value 0.45 |
|  | **Equivalised income group** |  |  |  |
| **2009/11** | Bottom Quintile (N=565) | Non-smoker (N=329)  Smoker (N=236) | 74 (243)  68 (162) | 83 (274)  80 (189) |
|  | 4^th^ Quintile (N=785) | Non-smoker (N=536)  Smoker (N=249) | 77 (415)  70 (174) | 85 (454)  83 (207) |
|  | 3^rd^ Quintile (N=759) | Non-smoker (N=565)  Smoker (N=194) | 81 (457)  64 (124) | 87 (493)  77 (150) |
|  | 2nd Quintile (N=850) | Non-smoker (N=666)  Smoker (N=184) | 90 (597)  73 (135) | 95 (630)  85 (156) |
|  | Top Quintile (N=1052) | Non-smoker (N=916)  Smoker (N=136) | 90 (827)  77 (105) | 95 (866)  89 (121) |
|  | Smoking*income |  | p value 0.16 | p value 0.25 |
| **2013/15** | Bottom Quintile (N=603) | Non-smoker (N=381)  Smoker (N=222) | 80 (306)  74 (165) | 86 (326)  89 (198) |
|  | 4^th^ Quintile (N=624) | Non-smoker (N=484)  Smoker (N=140) | 83 (400)  76 (106) | 89 (431)  85 (119) |
|  | 3^rd^ Quintile (N=725) | Non-smoker (N=588)  Smoker (N=137) | 87 (511)  77 (105) | 92 (543)  90 (123) |
|  | 2nd Quintile (N=855) | Non-smoker (N=715)  Smoker (N=140) | 92 (661)  85 (119) | 97 (691)  94 (131) |
|  | Top Quintile (N=849) | Non-smoker (N=751)  Smoker (N=98) | 91 (683)  84 (82) | 95 (719)  91 (89) |
|  | Smoking*income |  | p value 0.97 | p value 0.20 |
| **2017/19** | Bottom Quintile (N=697) | Non-smoker (N=512)  Smoker (N=185) | 84 (430)  78 (145) | 91 (464)  88 (162) |
|  | 4^th^ Quintile (N=761) | Non-smoker (N=594)  Smoker (N=167) | 83 (492)  63 (105) | 89 (530)  80 (133) |
|  | 3^rd^ Quintile (N=845) | Non-smoker (N=722)  Smoker (N=123) | 88 (632)  80 (99) | 93 (672)  86 (106) |
|  | 2nd Quintile (N=945) | Non-smoker (N=808)  Smoker (N=137) | 88 (714)  77 (106) | 96 (776)  91 (124) |
|  | Top Quintile (N=917) | Non-smoker (N=823)  Smoker (N=94) | 89 (734)  84 (79) | 96 (792)  93 (87) |
|  | Smoking*income |  | p value 0.09 | p value 0.47 |
|  | **Education group** |  |  |  |
| **2009/11** | No qualifications (N=707) | Non-smoker (N=461)  Smoker (N=246) | 66 (302)  60 (148) | 75 (346)  74 (183) |
|  | Other school level (N=261) | Non-smoker (N=193)  Smoker (N=68) | 67 (130)  62 (42) | 79 (152)  75 (51) |
|  | Standard grade or equivalent (N=768) | Non-smoker (N=501)  Smoker (N=267) | 87 (435)  74 (198) | 93 (468)  88 (235) |
|  | Higher grade or equivalent (N=669) | Non-smoker (N=504)  Smoker (N=165) | 87 (437)  74 (122) | 91 (460)  86 (142) |
|  | HNC/D or equivalent (N=454) | Non-smoker (N=358)  Smoker (N=96) | 90 (322)  72 (69) | 95 (340)  82 (79) |
|  | Degree or higher (N=1155) | Non-smoker (N=995)  Smoker (N=160) | 92 (913)  76 (122) | 96 (951)  84 (134) |
|  | Smoking*education |  | p value 0.33 | p value 0.12 |
| **2013/15** | No qualifications (N=524) | Non-smoker (N=373)  Smoker (N=151) | 73 (271)  66 (99) | 81 (301)  82 (124) |
|  | Other school level (N=194) | Non-smoker (N=151)  Smoker (N=43) | 75 (114)  70 (30) | 82 (124)  88 (38) |
|  | Standard grade or equivalent (N=695) | Non-smoker (N=471)  Smoker (N=224) | 89 (420)  82 (183) | 94 (442)  92 (205) |
|  | Higher grade or equivalent (N=607) | Non-smoker (N=496)  Smoker (N=112) | 92 (455)  79 (88) | 95 (473)  89 (99) |
|  | HNC/D or equivalent (N=455) | Non-smoker (N=379)  Smoker (N=76) | 92 (350)  82 (63) | 98 (370)  96 (73) |
|  | Degree or higher (N=1181) | Non-smoker (N=1049)  Smoker (N=132) | 91 (952)  86 (114) | 95 (1000)  92 (122) |
|  | Smoking*education |  | p value 0.81 | p value 0.33 |
| **2017/19** | No qualifications (N=591) | Non-smoker (N=430)  Smoker (N=161) | 74 (320)  64 (103) | 82 (354)  76 (123) |
|  | Other school level (N=228) | Non-smoker (N=191)  Smoker (N=37) | 79 (150)  62 (23) | 87 (166)  62 (23) |
|  | Standard grade or equivalent (N=705) | Non-smoker (N=542)  Smoker (N=163) | 89 (484)  83 (135) | 95 (517)  92 (150) |
|  | Higher grade or equivalent (N=640) | Non-smoker (N=525)  Smoker (N=115) | 87 (497)  79 (91) | 95 (497)  90 (103) |
|  | HNC/D or equivalent (N=512) | Non-smoker (N=408)  Smoker (N=104) | 88 (361)  74 (77) | 95 (386)  91 (95) |
|  | Degree or higher (N=1488) | Non-smoker (N=1362)  Smoker (N=126) | 90 (1227)  83 (105) | 96 (1313)  94 (119) |
|  | Smoking*education |  | p value 0.76 | p value 0.08 |
|  | **Occupation group (NS-SEC)** |  |  |  |
| **2009/11** | Routine occupations (N=565) | Non-smoker (N=340)  Smoker (N=225) | 75 (256)  61 (137) | 86 (291)  76 (173) |
|  | Semi-routine occupations (N=832) | Non-smoker (N=581)  Smoker (N=251) | 80 (464)  69 (172) | 86 (498)  82 (207) |
|  | Lower supervisory (N=412) | Non-smoker (N=282)  Smoker (N=130) | 79 (224)  72 (94) | 88 (247)  79 (103) |
|  | Small employers (N=283) | Non-smoker (N=208)  Smoker (N=75) | 83 (172)  73 (55) | 90 (187)  85 (64) |
|  | Intermediate occupations (N=548) | Non-smoker (N=432)  Smoker (N=116) | 85 (369)  80 (93) | 91 (394)  89 (103) |
|  | Lower managerial (N=968) | Non-smoker (N=807)  Smoker (N=161) | 90 (726)  70 (112) | 94 (760)  83 (134) |
|  | Higher managerial (N=406) | Non-smoker (N=363)  Smoker (N=43) | 91 (329)  88 (38) | 94 (340)  93 (40) |
|  | Smoking*occupation |  | p value 0.15 | p value 0.43 |
| **2013/15** | Routine occupations (N=525) | Non-smoker (N=356)  Smoker (N=169) | 84 (299)  74 (125) | 88 (314)  86 (145) |
|  | Semi-routine occupations (N=713) | Non-smoker (N=508)  Smoker (N=205) | 85 (433)  80 (163) | 91 (462)  91 (187) |
|  | Lower supervisory (N=269) | Non-smoker (N=201)  Smoker (N=68) | 85 (170)  63 (43) | 91 (183)  82 (56) |
|  | Small employers (N=305) | Non-smoker (N=247)  Smoker (N=58) | 87 (216)  79 (46) | 92 (228)  93 (54) |
|  | Intermediate occupations (N=535) | Non-smoker (N=443)  Smoker (N=92) | 88 (389)  84 (77) | 92 (409)  90 (83) |
|  | Lower managerial (N=896) | Non-smoker (N=783)  Smoker (N=113) | 92 (717)  84 (95) | 96 (754)  91 (103) |
|  | Higher managerial (N=415) | Non-smoker (N=381)  Smoker (N=34) | 88 (337)  85 (29) | 94 (359)  91 (31) |
|  | Smoking*occupation |  | p value 0.32 | p value 0.63 |
| **2017/19** | Routine occupations (N=584) | Non-smoker (N=417)  Smoker (N=167) | 81 (337)  74 (123) | 89 (371)  84 (140) |
|  | Semi-routine occupations (N=841) | Non-smoker (N=639)  Smoker (N=202) | 86 (547)  77 (155) | 93 (594)  84 (169) |
|  | Lower supervisory (N=336) | Non-smoker (N=265)  Smoker (N=71) | 86 (229)  63 (45) | 92 (245)  89 (63) |
|  | Small employers (N=323) | Non-smoker (N=253)  Smoker (N=70) | 84 (212)  70 (49) | 92 (244)  77 (54) |
|  | Intermediate occupations (N=524) | Non-smoker (N=462)  Smoker (N=62) | 88 (405)  79 (49) | 92 (427)  97 (60) |
|  | Lower managerial (N=1049) | Non-smoker (N=938)  Smoker (N=111) | 89 (839)  84 (93) | 96 (901)  95 (106) |
|  | Higher managerial (N=508) | Non-smoker (N=486)  Smoker (N=22) | 89 (433)  86 (19) | 95 (461)  95 (21) |
|  | Smoking*occupation |  | p value 0.38 | p value 0.03 |

Recent attendance = Attendance within 2 years; 5 year attendance = Attendance within 5 years; SIMD Scottish Index of Multiple Deprivation; N number; * Interaction test; HNC/D Higher National Certificates and Higher National Diplomas; NS-SEC National Statistics Socio-economic Classification; Weighted with combined-year survey weights.

**Supplementary Figure 1. Recent attendance of smokers and non-smokers by income group**


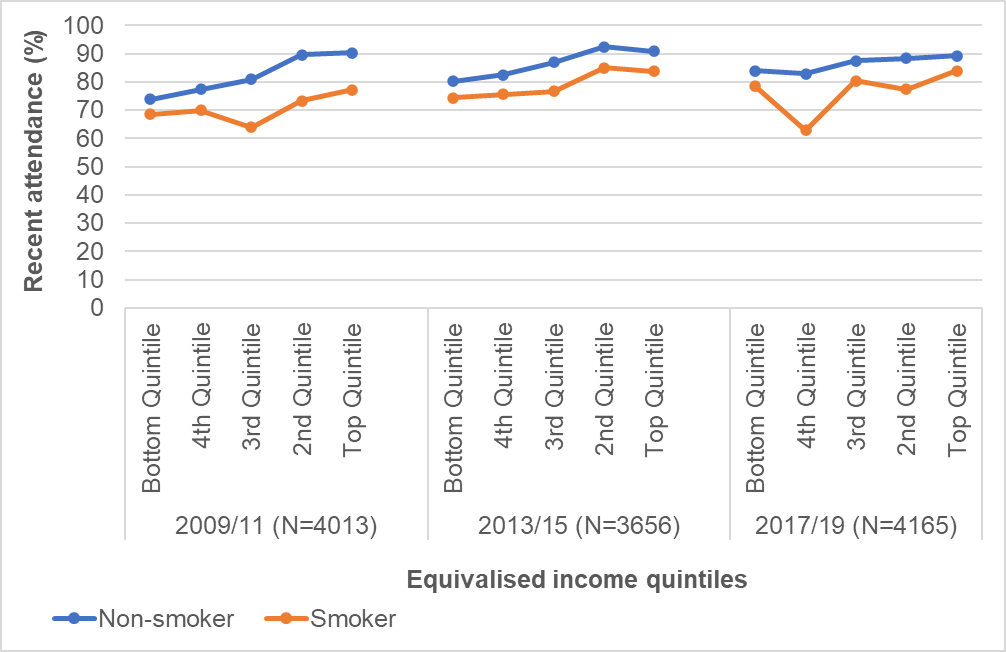
Recent attendance = Attendance within 2 years; N number

**Supplementary Figure 2 Recent attendance of smokers and non-smokers by education group**


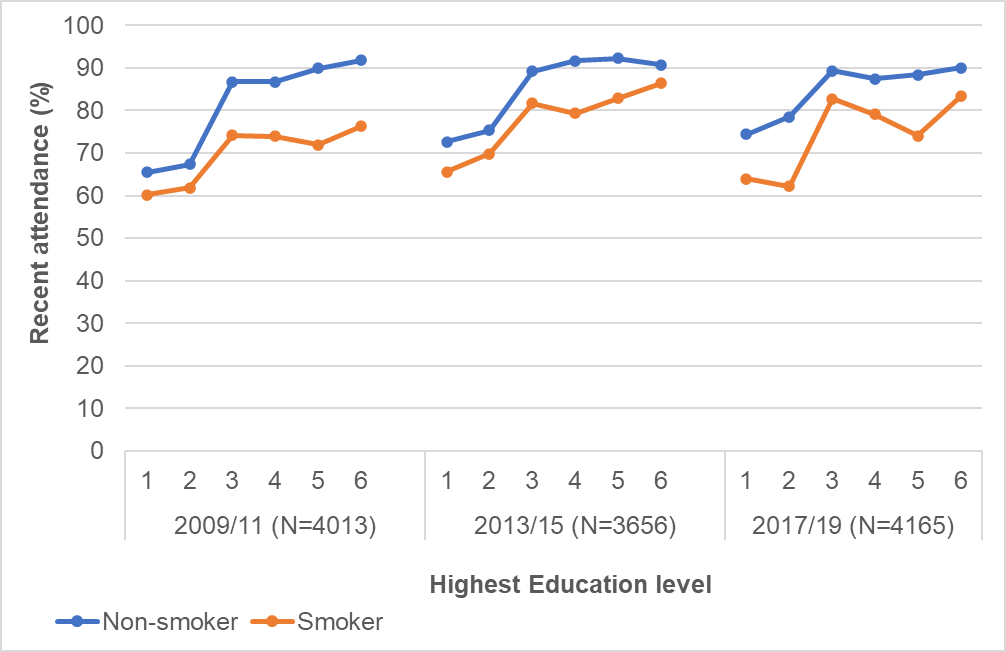


Recent attendance = Attendance within 2 years; N number; 1= No qualifications; 2= Other school level; 3= Standard grade/ GCSE or equivalent; 4= Higher grade/A level or equivalent; 5= HNC/D (Higher National Certificates and Higher National Diplomas) or equivalent; 6= Degree or higher.

|  | |  |
| --- | --- | --- |
|  |  |  |
|  | |  |
|  |  |  |
|  | | |
|  |  |  |
|  | | |
|  |  |  |
|  | | |
|  |  |  |
|  | |  |

**Supplementary Figure 3. Recent attendance of smokers and non-smokers by occupation group**


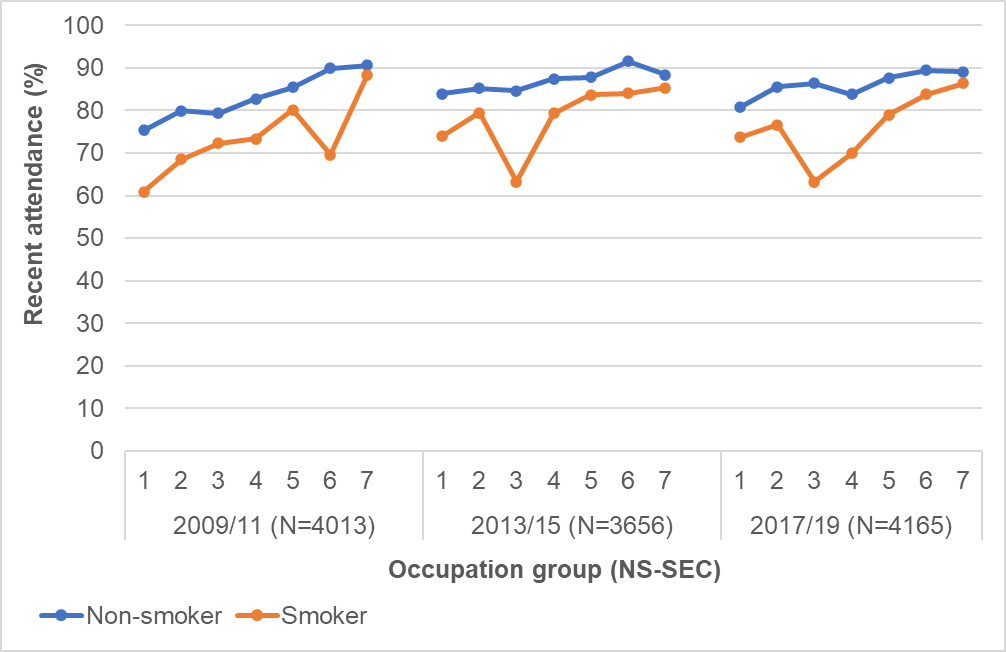


Recent attendance = Attendance within 2 years; N number; NS-SEC= National Statistics Socio-economic Classification; 1= Routine occupations; 2= Semi-routine occupations; 3= Lower supervisory and technical occupations; 4= Small employers and own account workers; 5= Intermediate occupations; 6= Lower managerial and professional occupations; 7= Higher managerial and professional occupations.

**Supplementary Figure 4.** **Risk Differences of Recent attendance of non-smokers relative to smokers**


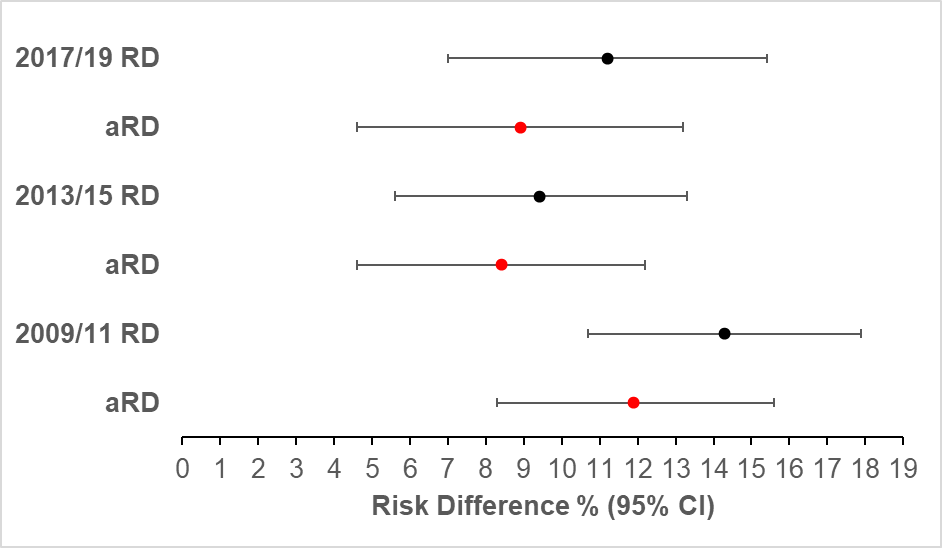


RD= Risk Difference; aRD= fully adjusted Risk Difference

**Supplementary Table 2. Risk Ratios (95% CI) for recent dental attendance comparing non-smokers to smokers**

| Scottish Health Survey Year  Recent attendance % (N) | Model 1  Univariable | Model 2  adjusted for age and sex | Model 3  adjusted as for Model 2 plus SIMD and survey year^a^ | Model 4  adjusted as for Model 3 plus individual SES measures^b^ |
| --- | --- | --- | --- | --- |
|  | RR (95% CI) | aRR (95% CI) | aRR (95% CI) | aRR (95% CI) |
| 2009/11 (N 4013) |  |  |  |  |
| Smoker (ref) 70 (701)  Non-smoker 84 (2539) | 1.0  1.20 (1.14, 1.27)  p value <0.0001 | 1.0  1.24 (1.18, 1.30)  p value <0.0001 | 1.0  1.20 (1.14, 1.26)  p value <0.0001 | 1.0  1.17 (1.11, 1.23)  p value <0.0001 |
| 2013/15 (N 3656) |  |  |  |  |
| Smoker (ref) 78 (577)  Non-smoker 88 (2562) | 1.0  1.12 (1.07, 1.18)  p value <0.0001 | 1.0  1.14 (1.09, 1.20)  p value <0.0001 | 1.0  1.13 (1.08, 1.18)  p value <0.0001 | 1.0  1.09 (1.04, 1.13)  p value <0.0001 |
| 2017/19 (N 4165) |  |  |  |  |
| Smoker (ref) 76 (534)  Non-smoker 87 (3003) | 1.0  1.14 (1.09, 1.21)  p value <0.0001 | 1.0  1.16 (1.10, 1.23)  p value <0.0001 | 1.0  1.14 (1.08, 1.20)  p value <0.0001 | 1.0  1.12 (1.06, 1.18)  p value <0.0001 |

Recent attendance = Attendance within 2 years; RR Risk Ratio; aRR Adjusted Risk Ratio; CI confidence interval; N number; c-index concordance statistic; ^a^survey year (individual year within each cohort), quintiles of the Scottish Index of Multiple Deprivation; ^b^education, occupation, income; Weighted with combined-year survey weights.

**Supplementary Table 3. Risk ratios (95% CI) for 5-year dental attendance comparing non-smokers to smokers**

| Scottish Health Survey Year  5 year attendance % (N) | Model 1  Univariable | Model 2  adjusted for age and sex | Model 3  adjusted as for Model 2 plus SIMD and survey year^a^ | Model 4  adjusted as for Model 3 plus individual SES measures^b^ |
| --- | --- | --- | --- | --- |
|  | RR (95% CI) | aRR (95% CI) | aRR (95% CI) | aRR (95% CI) |
| 2009/11 (N=4013) |  |  |  |  |
| Smoker (ref) 82 (824)  Non-smoker 90 (2717) | 1.0  1.10 (1.06, 1.14)  p value <0.0001 | 1.0  1.12 (1.08, 1.16)  p value <0.0001 | 1.0  1.10 (1.06, 1.14)  p value <0.0001 | 1.0  1.08 (1.04, 1.12)  p value <0.0001 |
| 2013/15 (N=3656) |  |  |  |  |
| Smoker (ref) 90 (660)  Non-smoker 93 (2710) | 1.0  1.04 (1.01, 1.07)  p value 0.01 | 1.0  1.05 (1.02, 1.08)  p value <0.0001 | 1.0  1.04 (1.01, 1.07)  p value 0.004 | 1.0  1.03 (1.00, 1.06)  p value 0.06 |
| 2017/19 (N=4165) |  |  |  |  |
| Smoker (ref) 87 (612)  Non-smoker 93 (3234) | 1.0  1.08 (1.04, 1.11)  p value <0.0001 | 1.0  1.10 (1.06, 1.13)  p value <0.0001 | 1.0  1.08 (1.04, 1.11)  p value <0.0001 | 1.0  1.06 (1.03, 1.10)  p value <0.0001 |

5 year attendance = Attendance within 5 years; RR Risk Ratio; aRR Adjusted Risk Ratio; CI confidence interval; N number; ^a^survey year (individual year within each cohort), quintiles of the Scottish Index of Multiple Deprivation; ^b^education, occupation, income. Weighted with combined-year survey weights

**Supplementary Table 4. Risk Differences (95% CI) for 5-year dental attendance comparing non-smokers to smokers**

| Scottish Health Survey Year  5 year attendance % (N) | Model 1  Univariable | Model 2  adjusted for age and sex | Model 3  adjusted as for Model 2 plus SIMD and survey year^a^ | Model 4  adjusted as for Model 3 plus individual SES measures^b^ |
| --- | --- | --- | --- | --- |
|  | RD % (95% CI) | aRD % (95% CI) | aRD % (95% CI) | aRD % (95% CI) |
| 2009/11 (N=4013) |  |  |  |  |
| Smoker (ref) 82 (824)  Non-smoker 90 (2717) | 7.9 (4.9, 10.9)  C-index 0.57  p value <0.0001 | 9.7 (6.8, 12.6)  C-index 0.74  p value <0.0001 | 8.0 (5.1, 11.0)  C-index 0.75  p value <0.0001 | 6.8 (3.8, 9.8)  C-index 0.77  p value <0.0001 |
| 2013/15 (N=3656) |  |  |  |  |
| Smoker (ref) 90 (660)  Non-smoker 93 (2710) | 3.3 (0.7, 5.9)  C-index 0.54  p value 0.01 | 4.8 (2.3, 7.4)  C-index 0.74  p value <0.0001 | 3.8 (1.2, 6.3)  C-index 0.75  p value 0.004 | 2.6 (0.1, 5.2)  C-index 0.77  p value 0.06 |
| 2017/19 (N=4165) |  |  |  |  |
| Smoker (ref) 87 (612)  Non-smoker 93 (3234) | 6.8 (3.8, 9.7)  C-index 0.56  p value <0.0001 | 8.3 (5.4, 11.2)  C-index 0.72  p value <0.0001 | 6.8 (3.9-9.7)  C-index 0.74  p value <0.0001 | 5.6 (2.7, 8.4)  C-index 0.77  p value <0.0001 |

5 year attendance = Attendance within 5 years; RD Risk Difference; aRD Adjusted Risk Difference; CI confidence interval; N number; C-index Concordance index; ^a^survey year (individual year within each cohort), quintiles of the Scottish Index of Multiple Deprivation; ^b^education, occupation, income; Weighted with combined-year survey weights.
